# Supplementary material for: Multidimensional analysis of screening results of deafness susceptibility genes in 3066 newborns of different altitudes and nationalities in Xining, Qinghai(ISRCTN89197487)
Source: PLoS One. 2026 Feb 24;21(2):e0342920. doi: 10.1371/journal.pone.0342920 (PMC12931801; doi:10.1371/journal.pone.0342920)
Supplement: S1 File — English version. (PDF) [file pone.0342920.s001.pdf]

Research Plan on the Application of Multidisciplinary Collaboration in Neonatal Deafness Gene Screening, Hearing Screening, and Hearing Intervention

I. Project Background and Research Significance

Neonatal hearing loss severely impacts language development, physical and mental health, and survival abilities, imposing a heavy burden on families and society. Early etiological diagnosis and intervention are crucial for reducing the harm of deafness. However, Qinghai Province previously lacked a large-scale, standardized combined screening system for neonatal deafness and a multidisciplinary collaborative diagnosis and treatment model, resulting in shortcomings in deafness prevention and control.

Relying on the scientific research and clinical resources of Qinghai University Affiliated Hospital (National Gene Detection Technology Application Demonstration Center), this project conducts the first large-sample multidisciplinary team (MDT) research on deafness screening and intervention in Qinghai Province. By integrating resources from multiple fields such as hearing screening, gene detection, clinical diagnosis, and genetic counseling, a localized standardized diagnosis and treatment system will be established. This not only clarifies the incidence of neonatal deafness and hotspots of gene mutations in Qinghai, providing a scientific basis for precise prevention and control and the formulation of medical insurance policies, but also achieves "early warning, early diagnosis, and early intervention" for deafness, significantly improving the regional level of hearing health, which holds important public health value and social significance.

II. Research Objectives

(I) Overall Objective

Establish a combined detection system of neonatal deafness gene screening and hearing screening in Qinghai Province, construct a mature multidisciplinary collaborative (MDT) diagnosis and treatment model, form a promotable plateau neonatal deafness prevention and control program, and reduce the disease burden of deafness.

(II) Specific Objectives

1. Complete combined deafness susceptibility gene and hearing screening for 3,000 neonates, and clarify the epidemiological characteristics of neonatal deafness/susceptibility gene carriers in Qinghai.

2. Establish a standardized follow-up mechanism to improve the follow-up compliance of high-risk infants with deafness, and realize the diagnosis of high-risk individuals within 7-14 days.

3. Form a local MDT hearing diagnosis team, and establish a full-process service model

integrating screening, diagnosis, intervention, and genetic counseling.

4. Cultivate a professional talent team, produce high-quality scientific research results, compile a Chinese-Tibetan bilingual publicity manual, and promote standardized prevention and control technologies.

III. Core Research Content

(I) Construction and Implementation of Combined Screening System

1. Screening Objects: All neonates born in Qinghai University Affiliated Hospital from January 2023 to December 2025; extended screening will be conducted for neonates with abnormal screening results and their parents (core families).

2. Screening Methods:

Hearing Screening: Adopt the DPOAE method; complete for normal neonates within 3 days after birth, and for high-risk infants in NICU before discharge under special circumstances.

Gene Screening: Collect 1mL of umbilical cord blood or heel blood, and detect 15 high-frequency mutation sites of 4 deafness susceptibility genes through gene chip.

3. Sample Management: Collect heel blood filter paper dried blood spots (3 pieces, 1mL blood volume each) in accordance with sterile sampling requirements, and send them to the central laboratory of the hospital at room temperature for testing.

(II) Research on Epidemiological Data and High-Risk Factors

1. Collect basic information, clinical data, gene detection results, and follow-up data of screening objects to establish a standardized database.

2. Analyze the incidence of neonatal deafness, carrier rate of susceptibility genes, and hotspots of gene mutations in Qinghai Province.

3. Analyze the high-risk factors affecting hearing loss, explore the core reasons for neonatal follow-up failure, and propose targeted measures to improve parental compliance.

(III) Establishment and Application of MDT Diagnosis and Treatment Model

1. Form an MDT team composed of multidisciplinary experts including Otorhinolaryngology, Pediatrics, Nursing, and Genetics, and clarify the responsibilities and collaborative processes of each discipline.

2. Provide one-stop services for high-risk infants with deafness and susceptibility gene carriers, including clinical diagnosis, hearing intervention, genetic counseling, and family guidance.

3. Formulate specifications for combined screening and intervention of neonatal deafness

in Qinghai, and establish a replicable and promotable localized model.

(IV) Science Popularization and Talent Training

1. Compile a Chinese-Tibetan bilingual "Plateau Neonatal Deafness Prevention and Treatment Publicity Manual" and carry out science popularization education for parents and primary medical institutions.
2. Cultivate 3 postgraduates and 3 further training professionals, and train 1-2 professional backbones per prefecture/county for prefectural and county-level medical institutions.

IV. Technical Scheme and Key Technologies

(I) Core Technical Methods

1. Hearing Screening Technology: DPOAE (Distortion Product Otoacoustic Emissions) detection, featuring rapidity, non-invasiveness, and high sensitivity, suitable for neonates.
2. Gene Detection Technology: Gene chip method, accurately detecting 4 deafness susceptibility genes (covering 15 high-frequency mutation sites) to achieve rapid etiological diagnosis.
3. MDT Collaboration Technology: Establish a linkage mechanism for multidisciplinary case discussion, referral, and follow-up to ensure seamless connection of the entire process from screening to diagnosis and intervention.

(II) Technical Difficulties and Solutions

1. Difficulty 1: Low neonatal follow-up rate and insufficient parental compliance. Solution: Establish a three-level follow-up network of "hospital-community-family", and improve cooperation through SMS reminders, special personnel docking, and science popularization education.
2. Difficulty 2: Impact of physiological characteristics of neonates in plateau areas on screening results. Solution: Optimize screening parameters, adjust detection standards in combination with plateau environmental characteristics, and ensure result accuracy.
3. Difficulty 3: Poor connection in multidisciplinary collaboration processes. Solution: Formulate a standardized collaboration manual, clarify the responsible subjects and time nodes of each link, and conduct regular team seminars.

(III) Innovations

1. Conduct the first large-sample epidemiological study on combined deafness gene and hearing screening in Qinghai Province, filling the regional data gap.
2. Establish an MDT diagnosis and treatment model for neonatal deafness adapted to plateau areas, realizing integrated services of "screening-diagnosis-intervention-genetic counseling".

3. Compile a Chinese-Tibetan bilingual publicity manual to meet the science popularization needs of ethnic minority areas and improve the coverage and accessibility of prevention and control.

V. Implementation Plan and Schedule

(I) Research Cycle

January 1, 2023 - December 31, 2025 (36 months)

(II) Annual Progress

Phase	Timeframe	Core Tasks
**Phase 1**	January 2023 - December 2023	Conduct combined screening for neonates of the year; establish a database; cultivate 1 postgraduate
**Phase 2**	January 2024 - December 2024	Conduct combined screening for neonates of the year; complete follow-up of screening objects from the previous year; cultivate 1 postgraduate; publish 1 paper in a Chinese core journal
**Phase 3**	January 2025 - December 2025	Conduct combined screening for neonates of the year; complete follow-up of screening objects from the previous two years; data collation and analysis; cultivate 1 postgraduate; publish 2 papers; compile a publicity manual; submit a final research report

VI. Risk Control and Response Measures

1. Risk 1: Unqualified sample collection affecting test results. Response: Formulate a standardized collection operation manual, conduct special training for collection personnel,

and perform quality review after collection.

2. Risk 2: Excessively high loss-to-follow-up rate during follow-up. Response: Establish multi-channel follow-up methods (phone calls, WeChat, community assistance), and provide transportation subsidies or free consulting services for follow-up objects to improve compliance.

3. Risk 3: Errors in detection technology. Response: The laboratory conducts regular quality control, participates in external proficiency testing, and ensures the normal operation of detection equipment and standardized personnel operations.

4. Risk 4: Delayed project progress. Response: Formulate monthly detailed task lists, assign special personnel to track progress, promptly coordinate and solve bottleneck problems, and reserve flexible time to respond to emergencies.
