# Supplementary material for: Multidimensional analysis of screening results of deafness susceptibility genes in 3066 newborns of different altitudes and nationalities in Xining, Qinghai(ISRCTN89197487)
Source: PLoS One. 2026 Feb 24;21(2):e0342920. doi: 10.1371/journal.pone.0342920 (PMC12931801; doi:10.1371/journal.pone.0342920)
Supplement: S1 Table — (DOCX) [file pone.0342920.s002.docx]

| **Genot(**  **DR:%)** | **Han** | **Tibetan** | **Hui** | **Mongolian** | **Middle-altitude areas** | **High-altitude areas** |
| --- | --- | --- | --- | --- | --- | --- |
| **GJB2** | 2.52 | 2.24 | 1.89 | 8.33 | 2.50 | 2.02 |
| **SLC26A4** | 2.47 | 0.93 | 2.43 | 0.00 | 2.54 | 1.08 |
| **Mt-12SrRNA** | 1.01 | 1.50 | 0.93 | 0.00 | 1.08 | 1.08 |
| **GJB3** | 0.05 | 0.00 | 0.00 | 0.00 | 0.04 | 0.00 |
