# Supplementary material for: Multidimensional analysis of screening results of deafness susceptibility genes in 3066 newborns of different altitudes and nationalities in Xining, Qinghai(ISRCTN89197487)
Source: PLoS One. 2026 Feb 24;21(2):e0342920. doi: 10.1371/journal.pone.0342920 (PMC12931801; doi:10.1371/journal.pone.0342920)
Supplement: Attachment — Submitted filename: Response to Reviewers.docx [file pone.0342920.s003.docx]

Dear Mr. Reynante Peña Balsamo and Mr. Joven Serdoncillo,

I hope this email finds you well. I am writing to confirm that I have completed the requested edits for my submission (Manuscript ID: PONE-D-25-52340; Title: Multidimensional analysis of screening results of deafness susceptibility genes in 3066 newborns of different altitudes and nationalities in Xining, Qinghai) and uploaded the relevant documents to the PLOS Editorial Manager system.

Specifically, the uploaded materials include:

The original ethical approval document for the study;

The clinical trial registration number (ISRCTN89197487) and the link to the online registration page (https://www.isrctn.com/ISRCTN89197487).

Please feel free to contact me if you need any further information or clarification. Thank you for your guidance and patience.

Best regards,

Yi Wang

E-mail: wegreatgroup@163.com
