# Supplementary material for: Multidimensional analysis of screening results of deafness susceptibility genes in 3066 newborns of different altitudes and nationalities in Xining, Qinghai(ISRCTN89197487)
Source: PLoS One. 2026 Feb 24;21(2):e0342920. doi: 10.1371/journal.pone.0342920 (PMC12931801; doi:10.1371/journal.pone.0342920)
Supplement: Attachment — Submitted filename: Response to Reviewers (3).docx [file pone.0342920.s004.docx]

**Cover Letter - Response to Reviewers**

**I. Responses to Additional Journal Requirements**

Format Compliance: All revisions have been made in strict accordance with the PLOS ONE formatting guidelines (including file naming conventions) provided in the template link.

Informed Consent Details: Detailed information on informed consent has been clearly stated in the "Materials and Methods" section of the manuscript as "with the written informed consent of the subjects' guardians". No further revisions are required, as the consent type (written) and applicability to minors (guardian consent) have been explicitly addressed both in the ethical statement and online submission information.

Clinical Trial Registration:

The ISRCTN registration number for this manuscript is ISRCTN89197487 (https://doi.org/10.1186/ISRCTN89197487). The registration platform name (ISRCTN) and registration number have been clearly indicated on the manuscript’s title page and in the online submission system.

a) The complete date range for subject recruitment and follow-up has been specified in the "Materials and Methods" section; no revisions are needed.

Funding Disclosure: The role of the funding source has been included in this cover letter as follows: "This study was supported by the Science and Technology Department of Qinghai Province, China (Project No.: 2023-SF-129). The funding source had no involvement in study design, data collection and analysis, decision to publish, or manuscript preparation."

Data Sharing: De-identified data has been uploaded to ISRCTN89197487, and access is available upon reasonable request from qualified researchers.

English Version of Dataset: We will supplement and submit the English version of the dataset as required (to comply with PLOS’s English-language publishing policy).

**II. Responses to General Author Comments**

Technical Rigor and Data Support for Conclusions: Both Reviewer 1 and Reviewer 2 confirmed the manuscript’s technical rigor and that data sufficiently supports the conclusions. No additional revisions are required.

Appropriateness of Statistical Analysis: Reviewer 1 approved the statistical analysis; revisions have been made to address Reviewer 2’s comment by adding 95% confidence intervals to the statistical section.

Transparency of Underlying Data:

Reviewer 1 confirmed data transparency. In response to Reviewer 2’s comment: De-identified basic subject information and outcome data have been uploaded to ISRCTN89197487 (https://doi.org/10.1186/ISRCTN89197487) and are accessible upon request.

Raw genetic sequencing data cannot be publicly shared due to regulatory restrictions: Qinghai Province (a high-altitude region with ethnic minorities in China) is a key area under the Chinese government’s genetic data management framework. Additionally, the Dean’s Office of the Affiliated Hospital of Qinghai University has prohibited the public disclosure of genetic data from Qinghai Province to protect ethnic and regional data security. We appreciate the journal’s understanding.

Clarity and Standard English: Reviewer 2 approved the language; specific errors identified by Reviewer 1 have been corrected to ensure the manuscript is clear, accurate, and free of ambiguity (consistent with PLOS ONE’s requirement for publication-ready English without editorial revisions).

**III. Responses to Specific Reviewer Comments**

Reviewer 1’s Comments:We sincerely appreciate Reviewer 1’s positive evaluation of our study (“a large-sample, interesting research exploring carrier frequencies of deafness variants across regions”). Regarding the comment on potential variant changes with extended follow-up and increased sample size: This valuable insight has been noted, and we will incorporate it into future research extensions to further validate the findings.

Reviewer 2’s Comments:

Excessive Figures: Non-essential figures (information sufficiently conveyable through text) have been moved to supplementary materials, with only core figures retained in the main text. Data on regions, variant types, frequencies, gender, ethnicity, and other variables have been summarized into tables for improved readability and comparability. Specifically, we have supplemented Table 4 after the references section of the manuscript to further enhance the readability and comparability of the manuscript.

Typographical Error in Table 3: The error (“CJB2” → “GJB2” and “GJB3”) has been rectified.

Regional Background for International Readers: To enhance international accessibility, clearer explanations of regional distribution and administrative divisions have been supplemented in the Introduction section, including geographic and demographic context relevant to non-Chinese readers.

Gene/Variant Selection Criteria and Limitations: The criteria for gene and variant selection have been explicitly specified. Additionally, the limitation that reported frequencies are restricted to the selected regions has been added and discussed in the Discussion section.

Figure Revisions:

Figures 1 and 2: Lines connecting unordered data points have been removed; 95% confidence intervals have been added for detection rate.

Figures 4-13, 14B, 15, 18, and similar figures: 95% confidence intervals have been uniformly added to incidence rates. Due to the large number of revised figure legends, explicit revision notes are included in the first few legends, with subsequent revisions implemented without redundant repetition.

Figure Merging: Redundant figures have been merged to convey equivalent information more concisely, optimizing manuscript structure.

IV. Review History and Author Identity

We confirm that neither author wishes to disclose their personal identity in the public review history (consistent with Reviewers 1 and 2’s preferences).

Yi Wang

Director of the Department of Otolaryngology at Peking Union Medical College Hospital, concurrently serving as Director of the Department of Otolaryngology at Qinghai University Affiliated Hospital

wegreatgroup@163.com

2025.12.17
