# Supplementary material for: Multidimensional analysis of screening results of deafness susceptibility genes in 3066 newborns of different altitudes and nationalities in Xining, Qinghai(ISRCTN89197487)
Source: PLoS One. 2026 Feb 24;21(2):e0342920. doi: 10.1371/journal.pone.0342920 (PMC12931801; doi:10.1371/journal.pone.0342920)
Supplement: Attachment — Submitted filename: To the Editorial Office of PLOS ONE2026.01.07.docx [file pone.0342920.s005.docx]

Dear Khem Descatamiento,

We are writing in response to your request regarding the data issues of our submission (PONE-D-25-52340R2). Due to the requirements of relevant local regulatory policies in Qinghai Province, the related data of this study cannot be shared publicly.

For researchers who meet the eligibility criteria for accessing confidential data, the data can be applied for from the Research Ethics Committee of the Affiliated Hospital of Qinghai University (contact number: 0971-6162033). We have also processed the Supporting Information file as requested, removing or anonymizing all personal identifying information (including specific dates) to ensure compliance with relevant regulations and participant consent.

Please let us know if any further clarification is needed. Thank you for your guidance.

Best regards,

Dr. Yi Wang
